# An Informative and Comprehensive Behavioral Characteristics Analysis Methodology of Android Application for Data Security in Brain-Machine Interfacing

**DOI:** 10.1155/2020/3658795

**Published:** 2020-03-10

**Authors:** Xin Su, Qingbo Gong, Yi Zheng, Xuchong Liu, Kuan-Ching Li

**Affiliations:** ^1^Hunan Provincial Key Laboratory of Network Investigational Technology, Hunan Police Academy, Changsha, China; ^2^Big Data Intelligence Police Hunan Provincial Engineering Research Center, Hunan Police Academy, Changsha, China; ^3^Zhejiang Economic Information Center, Hangzhou, China; ^4^Department of Computer Science and Information Engineering, Providence University, Taichung, Taiwan

## Abstract

Recently, brain-machine interfacing is very popular that link humans and artificial devices through brain signals which lead to corresponding mobile application as supplementary. The Android platform has developed rapidly because of its good user experience and openness. Meanwhile, these characteristics of this platform, which cause the amazing pace of Android malware, pose a great threat to this platform and data correction during signal transmission of brain-machine interfacing. Many previous works employ various behavioral characteristics to analyze Android application (or app) and detect Android malware to protect signal data secure. However, with the development of Android app, category of Android app tends to be diverse, and the Android malware behavior tends to be complex. This situation makes existing Android malware detections complicated and inefficient. In this paper, we propose a broad analysis, gathering as many behavior characteristics of an app as possible and compare these behavior characteristics in several metrics. First, we extract static and dynamic behavioral characteristic from Android app in an automatic manner. Second, we explain the decision we made in each kind of behavioral characteristic we choose for Android app analysis and Android malware detection. Third, we design a detailed experiment, which compare the efficiency of each kind of behavior characteristic in different aspects. The results of experiment also show Android malware detection performance of these behavior characteristics combine with well-known machine learning algorithms.

## 1. Introduction

Brain-machine interfaces (BMIs) are a communication technology that link humans and artificial devices through brain signals. Many mobile applications (or apps) are designed as an assistant tool to improve BMIs availability. Android is currently the most popular smart-mobile device platform in the world, occupying about 85% of market share. As of now, there are nearly 3 million Android applications available for downloading from Android official market. The rapid growth of Android and its apps have led to an increased amount of malware. Android malware has resulted in immense losses and security threats of individuals and businesses, such as data leak during transmission [1]. Developing techniques to analyze and detect Android malware and protect the signal data do not falsify during transmission has become an emergent critical issue.

Traditional Android malware detection approaches focus on signature matching, which is simple and efficiency on detecting known Android malware. However, more and more zero-day malware sprang up all over popular Android markets, which make this approach disable to detect them with high accuracy. Some researchers employ behavioral characteristic with machine learning algorithm to solve limitations of signature matching approach. These kinds of works can divide into two categories: static behavioral characteristic analysis [2–8] and dynamic behavioral characteristic analysis [9, 10]. For example, Li et al. [2], DroidAPIMiner [3], APK Auditor [4], and Drebin [5] focus on extracting static behavioral characteristics from Android app and then combine them with well-known classification algorithms to detect Android malicious app. However, this kind of approach cannot detect malware with code obfuscation and also cannot capture behavioral characteristics when running the app. Therefore, some researchers employ dynamic behavioral characteristics with machine learning algorithm to detect Android malware. For example, Enck et al. [9] define several privacy information source simultaneously employing dynamic tainting analysis. Su et al. [10] propose an approach which capture HTTP traffic of Android app running to detect Android malware. However, this kind of approach needs to modify code with Android OS version update which lack of scalability.

Based on these previous research works, many kinds of behavioral characteristics have been used to analyze and detect Android malware. However, these are three problems still not solved. First, these works choose several kinds of behavioral characteristics to analyze Android malware but do not explain decisions on these behavioral characteristics. Second, these works do not explain whether behavioral characteristics of Android malware change over time. Third, which kind of behavioral characteristics plays important role during Android malware detection is not illustrated in these works.

To address these problems, we propose a broad and efficient Android malware behavioral characteristics analysis approach under a real-world dataset. First, we extract 11 different static behavioral characteristics and 12 different dynamic behavioral characteristics from each Android app. Second, the proposed method provides an explainable detection, and we explain the reason why we select these kinds of behavioral characteristics. Third, we fed these kinds of behavioral characteristics into several well-known machine learning models to observe which kind of behavioral characteristics would be efficient during Android malware detection. Finally, we compare behavioral characteristics of Android malware change over time.

Based on above description, we summarize the main contributions of this paper as follows:

- We introduce an analysis approach combining static and dynamic behavioral characteristics that is capable of depicting Android malware with comprehensive and accuracy, which is able to provide a high-quality behavioral characteristic dataset for Android malware detection.
- We provide an explainable analysis for each kind of behavioral characteristics we extracted. This analysis could explicit illustrate each kind of behavioral characteristics playing the role of Android malware detection.
- In the experiment, we analyze the detection results of each kind of behavioral characteristic and combination of behavioral characteristics. Moreover, we also compare behavioral characteristics of Android malware collected from different periods of time and observe whether behavioral characteristics would change with time change.

The reminder of this paper is organized as follows: the related works have been described in Section 2. We explain reason for each kind of extracted behavioral characteristics in Section 3. The experiment of this paper is shown in Section 4. Finally, we summarize the proposed approach in Section 5.

## 2. Related Work

There are many previous research works paying attention on Android malware detection and behavioral characteristic analysis. In this section, we divide these approaches into two categories, namely, static analysis and dynamic analysis.

### 2.1. Static Analysis

The first category of approach for detecting Android malware was inspired by static program analysis. Several methods have been proposed that statically inspect Android apps and disassemble their code. For example, static approach includes analyzing permission requests for app installation [11] and signature-based detection [12].

The static analysis approach focuses on Android malware analysis based on the static behavioral characteristic, which disassembles the Android app installation file (.apk file) into configuration file and source code. Enck et al. [13] decompile a set of popular Android apps back into dex file and then identify untrusted problems. Yang et al. [14] present a static analysis method called AppContext, which is able to distinguish Android benign app and malware. This work uses contexts that trigger security-sensitive to classify the Android apps. Zhu et al. [15] present an approach that collects requested permission, sensitive API, and system event as behavioral characteristic and use the classification algorithm to build a model for Android malware detection. Mehtab et al. [16] proposes a framework, AdDroid, for analyzing and detecting malicious behavior in Android applications based on various combinations of dynamic behaviors such as accessing network, uploading a file to a remote server, or installing another package on the device. AdDroid employs an ensemble-based machine learning technique where Adaboost is combined with traditional classifiers in order to train a model found on static analysis of Android applications that is capable of recognizing malicious applications. Xie and Li [17] proposed an Android malware detection model based on Bagging-based SVM and static features (permission, intent, and component) extracted from *AndroidManifest.xml*. This work first proposed the *IG-Relief hybrid* selection algorithm to reduce the dimension of the dataset and then used a Bagging-based SVM ensemble classifier trained by the multiple balanced datasets to detect Android malware.

The proposed approach of this paper has been related to the above works; however, our work is different from them. First, we extract far more behavioral characteristics to depict Android benign app and malware comprehensively. Second, we explain reasons for the extracted behavioral characteristics for the purpose of Android malware detection.

### 2.2. Dynamic Analysis

Unlike static analysis approach, this kind of analysis approach focuses on capturing runtime behavior from the Android app while running on an Android emulator or real devices, for example, dynamic taint analysis [18] and dynamic behavior-based detection [19, 20].

DroidScope [18] allows dynamically monitoring several apps in a protected environment, where the former focuses on taint analysis and the latter enables introspection at different layers of the platform. While both systems provide detailed information about the behavior of apps, they are technically too involved to be deployed on smartphones and detect malware directly. Saracino et al. [19] propose a framework (MADAM) for Android malware detection which monitors apps at the kernel and user level. The MADAM detects system calls at the kernel level and user activity/idleness at the user level to capture the app behavior. Crowdroid [20] is another behavior-based malware detection approach for Android that uses system calls and machine learning techniques. Crowdroid collects information about system calls through a community of users. A lightweight app, installed in the user's devices, monitors system calls (frequency) of running apps and sends them to a centralized server. The server produces feature vectors and applies a K-means clustering to classify the apps as malware or benign app. Hou et al. [21] propose a novel dynamic analysis method named component traversal that can automatically execute the code routines of each given Android app as completely as possible. Ali et al. [22] introduce the use of Fuzzy C-means clustering combined with the generated network traffic in Android malware detection. The features selected in this work were extracted from the network traffic and then were used in Fuzzy C-means clustering algorithm.

However, dynamic taint analysis approaches need to modify OS code, which lacks scalability. Other dynamic analysis approaches focus on few behavioral characteristics, such as network traffic and system call, and cannot depict the comprehensive dynamic behavior. In our work, we extract 12 dynamic behavioral characteristics including network traffic and system call for analysis and we not need modify OS code to track data flow during Android app running.

## 3. Behavioral Characteristic Analysis

In this section, we divide behavioral characteristics of Android app into static and dynamic and extract 11 different kinds of them, respectively. Then, we give a deep analysis of each kind of extracted behavioral characteristic and explain them in detail.

### 3.1. Behavioral Characteristic Extraction

#### 3.1.1. Static Behavioral Characteristic Extraction

**Figure 1:**
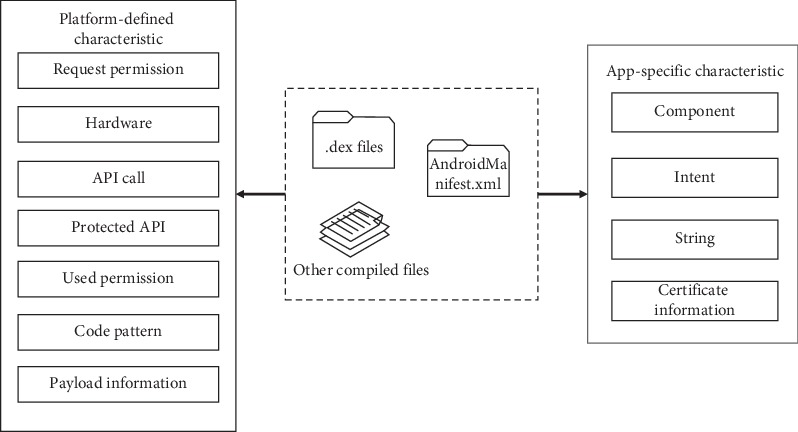
Architecture of the static behavioral characteristic extraction.

Our static behavioral characteristic extraction focuses on *AndroidManifest.xml* and *class.dex*, and the Android apps were systematically profiled into six types: requested permission, used permission, sensitive API call, action, app component, and intent. Therefore, we extract these types of features from the crawled Android apps, and these types of behavioral characteristics are comprehensive yet a unique representation of Android apps that help determine the typical indications of malicious activity. The detailed extraction process is shown in Figure 1.

To obtain *AndroidManifest.xml* and *class.dex*, we need to decompress the *.APK* files by *apktool* which is a tool for reverse engineering Android apk files [23]. After decompressing, we start to extracting behavioral characteristic from *AndroidManifest.xml* and *class.dex*. The *AndroidManifest.xml* file contains several configuration information of Android app, such as requested permission and app component. We parse this file by employing *AXMLPrinter2* and *TinyXml* and then extract the behavioral characteristic of requested permission, intent, action, and app component. The *class.dex* file is responsible for storing *Dalvik byte code* which can be converted to *smali code* [24] for better behavioral characteristic extraction. Used permission and sensitive API call can be extracted from *smali code*. In [25], API call and requested permission are matched to discover which permission is used. Therefore, we can obtain the used permission by extracting the API call. We define several customized extraction rules in xml files. For instance, we focus on the APIs provided by the Android framework, so that we can define a rule as List 1 shows.

In Listing 1, the value of regex is defined in regular expression. According to this rule, the decoder can extract APIs such as *android.telephony.TelephonyManager.getSimSerialNumber* from *smali code*. Other behavioral characteristics can be extracted by the similar rules. The node multiMatch indicates whether we want the regex to be matched more than once or not.

#### 3.1.2. Dynamic Behavioral Characteristic Extraction

Unlike static behavioral characteristics, dynamic behavioral characteristics are able to depict running behaviors of the Android app. To extract this kind of behavioral characteristic, we need to install an Android app and operate it in an Android smartphone or emulator. Therefore, we design an automatic tool to install and operate the Android app.

**Figure 2:**
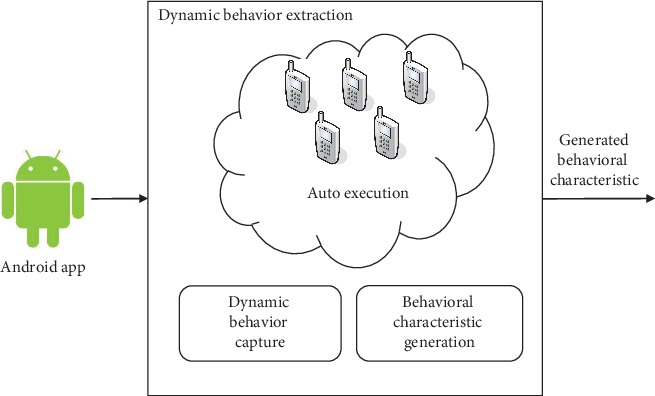
Architecture of dynamic behavioral characteristic extraction.

From Figure 2, we find that the proposed approach consists of three modules: first is app execution, which automatically executes Android apps on several real smartphones or Android emulators and outputs the captured dynamic behavioral characteristics (e.g. network traffic and system call) that are generated during execution. This part is also configurable by choosing different rules, e.g., composition of Android apps, execution duration, and execution behavior. The second part is responsible for receiving the captured dynamic behavioral characteristic as input, and then extracting the packet, flow, and system call. The third part is the dynamic behavioral characteristic generator which reads configurable file and generates a dynamic behavioral characteristic for high-level study.

### 3.2. Behavioral Characteristic Analysis

#### 3.2.1. Static Analysis

In this section, we explain the behavioral characteristic of the Android platform we mainly used in profiling applications by static analysis. Our static analysis consists of several parts which focus on the Android manifest file and disassembled dex code of the app. Every app developed for Android must include a manifest file called AndroidManifest.xml which provides data supporting the installation and later execution of the application. This kind of file contains requested permission, app component, intent, and action.

- App component: a typical Android app contains *Activity*, *Service*, *Content provider*, and *Broadcast receiver* and defines these components in the Android configuration file for development purpose. Some malware in the same family may share the same name of the app component.
- Intent: this component is a lightweight message delivery mechanism, which can delivery messages among different components inside an Android app and between different apps. We found that some specific intents are more frequently defined in the Android malicious app than in the benign one, because Android malware need to delivery particular messages during the launch of malicious activities.
- Requested permission: an Android app would require predefined permission to access corresponding resources. For example, some of malicious apps would request the *READ_SMS* permission to read SMS messages in background. Therefore, requested permission is a useful feature for distinguishing Android malware.
- Hardware: smartphone contains several hardware, such as camera and Bluetooth. If an app wants to request these kinds of hardware, the developer needs to declare them in the Android configuration file. Some malwares would utilize this feature to request hardware to monitor the user, such as recording monitor.

The second part is byte-code reverse from the Android app. This kind of file includes API call, used permission, code pattern, and string. We will illustrate these behavioral characteristics in details as follows.

- API call: application programming interface (or API) is a set of system interface for application call. The API call is able to depict behaviors of Android app for identifying sensitive operations. Android malware may frequently invoke some certain functions to obtain sensitive data from device. Therefore, extracting this kind of feature could be helpful to detect Android malware.
- Protected API: some API could access the sensitive data or resource of smartphone. We define such kind of API as protected API, which is important for smartphone data security. Based on our observation, a majority of Android malware would invoke such kind of API to leak sensitive data from smartphone. For example, *getSubscriberId* and *getDeviceId* may read *IMSI* and *IMEI* from the smartphone.
- Used permission: this kind of feature reflects the permission used when Android app running, which can depict the behavior more precisely than *Requested Permission.* Based on the mapping relationship of API-permission and Intent-permission, we can obtain used permission from API calls and intent information [26].
- Code pattern: this kind of feature can reflect whether an Android app loads external executable files when it is running. We extract this feature from disassemble code of Android app, such as dynamic load dex files and Linux commands. For example, some Android malware would obtain permission to operate files by executing the *chmod 777* command.
- String: this kind of feature contains URL, IP address, and file path extracted from the disassembled code. Some malwares would leak privacy data by network. For example, a malware family called *Basebridge* leaked sensitive information to http://b4.7755.org:8088 [27].

Except Android app configuration file and disassembled code, we also extract behavioral characteristic from certification and file suffix.

- Certificate information: this behavioral characteristic indicates the author of the Android app. The app developer uses a secret key to sign the apk file when the app is released. The certificate information contains several developer information such as country, e-mail, and organization, which could differentiate the developers. Kang et al. detect and classify Android malware using this information [28].
- Payload information: this feature indicates the file category contained in the apk file. Some malwares may contain extra dex files in their install file and load them when the app is running.

After 11 kinds of static behavioral characteristics have been explained, we conduct a depth analysis of these characteristics. We found that the extracted characteristics can be divided into two categories based on their providers. The first category is the *platform-defined characteristic*, which represents these kinds of behavioral characteristics provided by the Android platform. Another category is the *app-specific characteristic*, which represents behavioral characteristic defined by the Android app developer. Based on this categorization, we can depict the behavioral characteristic of Android malware in a fine-grained manner. Tables 1 and 2 show several examples for this categorization. The Android app data set we extract and category behavioral characteristic are described in Section 4.

#### 3.2.2. Dynamic Analysis

Dynamic analysis represents behavioral characteristic captured during the Android app running time, which mainly includes network traffic and system call. Network traffic mainly depicts behaviors when the Android app accesses Internet. We divide this kind of behavior into three kinds of behavioral characteristics:

- Quantitative behavioral characteristic: this category measures and compares the volumes of traffic across malware and benign apps. When the malware communicates with the malicious servers, they request update commands and leak private information with a fixed format. Also, the malware do not generate large traffic volumes to avoid detection by antivirus scanners or intrusion detection systems. Therefore, a malware trace might contain many flows with similar traffic size.
- Timing-based behavioral characteristic: the second type of feature category is the time-based behavioral characteristics, which try to capture the duration of activity of the Android app.
- Semantic behavioral characteristic: more than 90% of apps run over the HTTP protocol and that 93% of malware samples use HTTP to receive commands from their C&C servers that can be found in the collected Android apps. Thus, considering this scenario, the network behavior can be correlated to the semantics of the different HTTP requests and responses. The network behavior changes with respect to the HTTP method, contacted hosts, URL paths or queries, and so on.

The Android Operation System is based on Linux kernel. In Linux, a system call is how a program requests a service from the operating systems kernel. System calls provide useful functions to application programs like network communication, file management, or process-related operations. When an app from the user space makes a request to the Operation System, the request goes through glibc library, system call interface, kernel, and finally to the hardware. Functions like getpid(), open(), read(), chmod(), and socket() are some of the functions that glibc provides for apps to invoke a system call.

## 4. Experiment

In this section, we focus on evaluating efficiency of the extracted behavioral characteristic. To achieve this, we conduct the following experiments:

- Performance of single behavioral characteristic: in this experiment, we evaluate the performance of each kind of behavioral characteristic and observe which kind of behavioral characteristic combined with machine learning algorithm could be more useful in Android malware detection.
- Performance of behavioral characteristic combination: in this experiment, we combine several behavioral characteristics into different groups based on their provider. Then, which combination could be more efficient during Android malware detection is evaluated.
- Behavioral characteristic persistence: in this experiment, we evaluate the persistence of static and dynamic behavioral characteristics on classification performance with two datasets collected in different time periods.

### 4.1. Dataset

Before evaluating our proposed approach, we first introduce the dataset we used. The dataset consists of three parts: first dataset consists of Android app obtained from the official market and covers the most popular Android app from each category. We consider this kind of dataset as a benign app because of strict audit mechanism of the official market. Second dataset consists of several well-known malware dataset, such as Drebin [5], Android Malware Genome Project [29], and the Contagio Community [30]. The third dataset consists of Android apps downloaded from several unofficial markets, and we consider this kind of dataset as an unknown type of Android app. At last, we totally get 3,986 Android benign apps, 3,986 Android malicious apps, and 1,515 unknown Android apps, and extract their behavioral characteristics storage in the database.

### 4.2. Performance of Single Behavioral Characteristic

In Section 3, we extract 11 static behavioral characteristics and 12 dynamic behavioral characteristics from the Android app. However, single dynamic behavioral characteristics are common for the Android benign app and malware. Therefore, we choose single behavioral characteristics to classify the Android benign app and malware.  Table 3 shows the performance of Android malware classification based on a single kind of static behavioral characteristics.

#### 4.2.1. Static Behavioral Characteristic

From Table 3, we find most of single behavioral characteristic cannot achieve high performance, and the performance is differentiated by combining with different machine learning algorithms. *Requested permission* combined with *Random forest* is able to achieve the highest performance among all kinds of behavioral characteristics. In summary, the results of this experiment demonstrate that single behavioral characteristic cannot classify Android benign app and malware well.

#### 4.2.2. Dynamic Behavioral Characteristic

**Figure 3:**
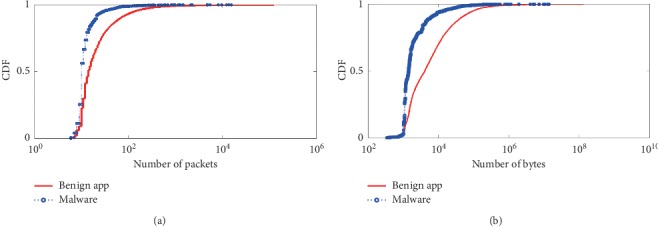
Comparison of the numeric aggregates of transfer size. (a) Number of packets. (b) Number of bytes.

**Figure 4:**
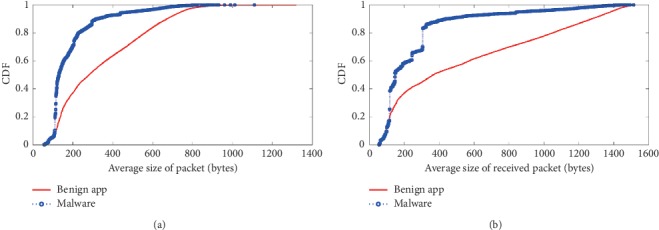
Comparison of the numeric aggregates of inward flow. (a) Number of received packets. (b) Average size of received packets.

**Figure 5:**
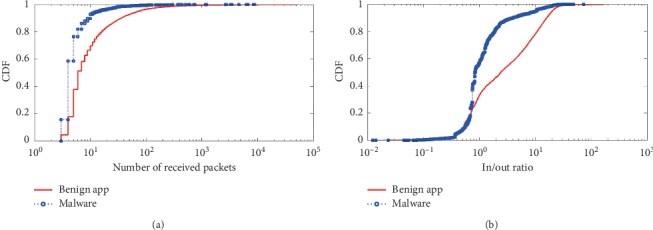
Data sizes and relative traffic ratios of flows. (a) Average size of packets. (b) In/out ratio.

Next, we will compare each dynamic behavioral characteristic between Android benign app and malware. The first category is the *quantitative behavioral characteristic* which includes the *number of packets*, *number of bytes*, *number of received packets*, *average bytes of received packets*, *average size of packets*, and *in/out ratio*. The detail results of comparison are shown in Figures 3–5.

In Figure 3(a), 80% of malware flows containing about 10 packets or less have been observed; only 30% of benign app flows achieve this number. In Figure 3(b), 80% of malware flow size reaching 1000 bytes or less has been observed; only 30% benign app flow size achieves this number. Because benign apps have rich functionality, their network activities include, text chat, videos, and image downloading. Therefore, these network activities are expected to have a variable number of packets due to the variable size of the data involved. On the contrary, malware focuses on sending out private data out, which is usually in standard size regardless of the smartphone in use, and hence, it is expected that the number of packets per flow is similar across the multiple malicious apps.

In Figure 4(a), benign app flows contain more received packets per flow than malware. Also, Figure 4(b) shows the average packet size in each flow. The relative difference in packet sizes is clear from these results. Because benign apps may receive large size files from servers, due to the limitation of a packet length, the large size file is split into several segments, thereby increasing the number of received packets; whereas in malware apps, the received command packets are usually small-sized packets.

In Figure 5(a), almost 50% of malware packet sizes are in the range from 101 bytes to 200 byte, and less than 30% of benign app packets sizes fall in this range. In Figure 5(b), for benign apps, note that about 20% of the ratios of the incoming and outgoing traffic are lesser than 1, which shows that this traffic contains more sent data than received data. In benign apps, the packet size is not constrained as the user can download or upload data of any size. For malware apps, the command packets typically have a smaller size due to the compact nature of the Botnet protocol communication.

**Figure 6:**
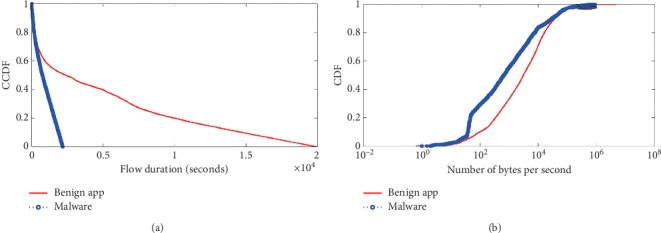
Flow duration comparison. (a) Flow duration. (b) Number of bytes per second.

The second category of this kind behavioral characteristic is the *Timing-Based behavioral characteristic* which includes *flow duration* and *the number of bytes per second*. The detail results of comparison are shown in Figure 6.

The duration of flow is typically the TCP session length, which represents the amount of time an app requires to conduct its network functions with its destination server.  Figure 6(a) shows a CCDF plot of the HTTP flow duration in benign apps, malware apps and ad libraries. We notice that, for benign app and ad libraries, more than 40% flows have a duration shorter than 2 seconds. This is because many flows in benign apps and ad libraries only transfer small data like text or small image files for which the duration is short. This figure also shows that benign apps account for a larger proportion of long duration flows.  Figure 6(b) shows the CCDF of the number of bytes per second in the benign app, malware app, and ad library. There is a clear gap that can be observed, i.e., 70 bytes/s as compared with 1200 bytes/s, between the benign app and malware, respectively, which demonstrates that the malware communication is lightweight, stealthy, and ends in a short time period.

**Figure 7:**
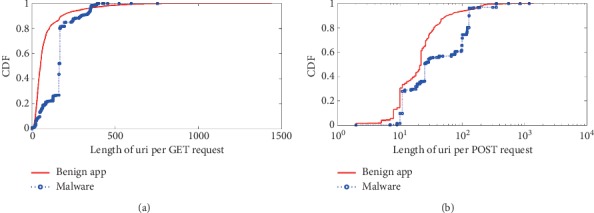
URI length of HTTP request. The length of URI per (a) GET request and (b) POST request.

**Figure 8:**
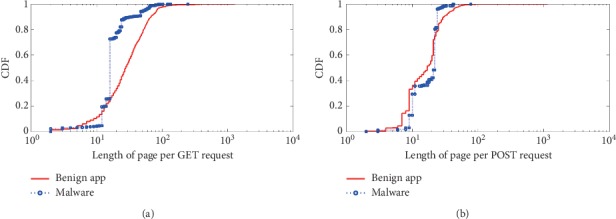
Page length of HTTP request. The length of page per (a) GET request and (b) POST request.

**Figure 9:**
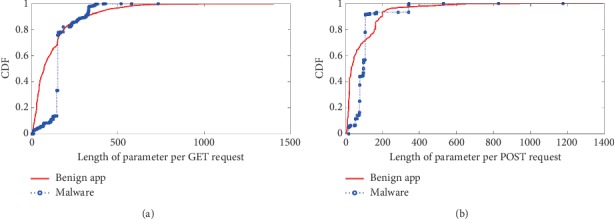
Parameter length of HTTP request. Length of parameter per (a) GET request and (b) POST request.

The third category is the *semantic behavioral characteristic* which includes *length of URI per GET/POST request*, *length of page per GET/POST request*, and *length of parameter per GET/POST request*. The detail results of comparison are shown in Figures 7–9.

The length of URI per GET/POST request shows the number of resources requested by the app. Benign apps may request various kinds of files, whereas malware usually request commands like update or leak private data out in a fixed format.

The length of page represents the paths visited by the app to obtain the resources, and typically, the same HTTP request contains more than one resource path. Benign apps usually request multiple resources as they try to maximize the user experience, and on the other hand, malicious apps request a small number of resources.

The GET/POST parameter is a query string, which is the part of a uniform resource locator (URL) that contains data to be passed to servers. Because benign apps have various types, and they may send requests to servers with variable parameter formats. However, the malware asks commands like update and leak private data out with fixed parameter format, and usually the parameter lengths are fixed within a small statistical threshold.

In summary, single behavioral characteristic of network traffic is able to distinguish the Android benign app and malware partly. However, the Android malware can forge such single behavioral characteristic easy to avoid detection. Therefore, we conduct the second experiment and utilize the characteristic combination to depict Android malware behaviors.

### 4.3. Performance of Behavioral Characteristic Combination

We have evaluated a single kind of behavioral characteristic that cannot obtain good performance during the Android malware classification in Section 4.2. In this experiment, we try to combine several kinds of behavioral characteristics based on their provider to evaluate their performance. To achieve this goal, we divide the 11 static behavioral characteristics into two sets, namely, *platform-defined* and *app-specific*. *Platform-defined* represents the behavioral characteristic defined by the Android platform, and *app-specific* represents appSpecific defined by each Android app. Meanwhile, we also divide 12 dynamic behavioral characteristics into 4 sets, namely, *quantitative behavioral characteristic*, *timing-based behavioral characteristic*, *semantic behavioral characteristic*, and *system call*. Moreover, we combine all static and dynamic behavioral characteristics into two behavioral characteristic sets to evaluate its performance, respectively. The detailed results are shown in Tables 4 and 5.

From Table 4, we can find that employing the behavioral characteristic of *app-specific* can obtain better performance than *platform-defined*, because behavioral characteristic of *app-specific* is defined by the Android app which can depict the unique behavioral characteristic. Moreover, combining both kinds of behavioral characteristics can obtain the best performance among the three kinds of behavioral characteristics. This result demonstrates that the extract comprehensive behavioral characteristic can be helpful for the Android malware classification.

From Table 5, we can find that any kind of dynamic behavioral characteristic cannot obtain a good performance, because these kinds of behavioral characteristics may be overlapped by the Android benign app and malware. However, combining these kinds of behavioral characteristics can achieve better performance, due to its uniqueness.

### 4.4. Behavioral Characteristic Persistence

For evaluating the behavioral characteristic persistence of Android app over time, this experiment first divides our Android app dataset into two categories based on different periods of time. The detailed information is listed in Table 6.

**Figure 10:**
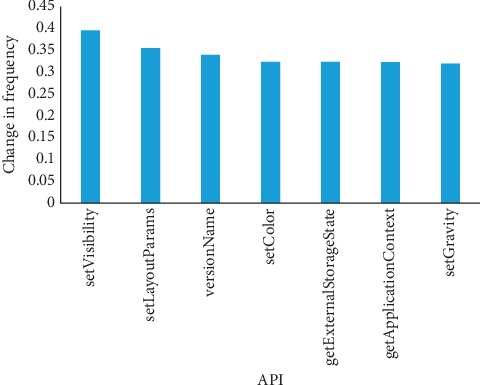
API call variation frequencies between *Pass* and *New* datasets.

From Table 6, the kind of *Pass* dataset represents these apps released before 2015, and *New* dataset represents these apps released after 2015. In this experiment, we choose three kinds of static behavioral characteristics as an example to evaluate persistence of behavioral characteristics. The behavioral characteristic of *API* persistence is shown in Figure 10.

**Figure 11:**
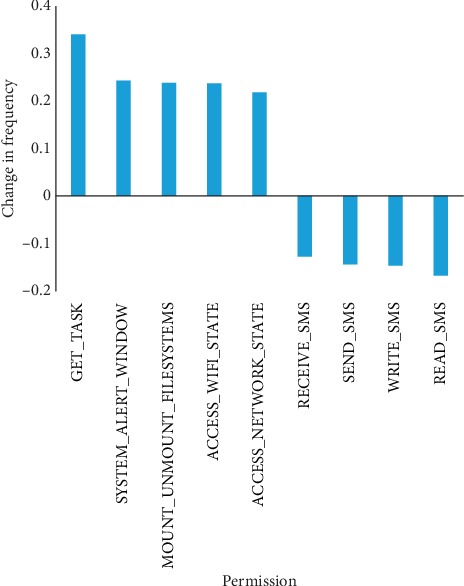
Permission variation frequencies between *Pass* and *New* datasets.

From this figure, we find that Android malware from *New* dataset calls more APIs of UI elements than the *Pass* dataset, such as visibility setting, layout parameter setting, and color. Because majority of Android malwares embed malicious code into the benign app, these benign apps contain more UI elements with change of time. Another reason for this situation is that Android malware needs to avoid detection and pretends as a benign app by design similar to UI.  Figure 11 shows the variation of permission.

**Figure 12:**
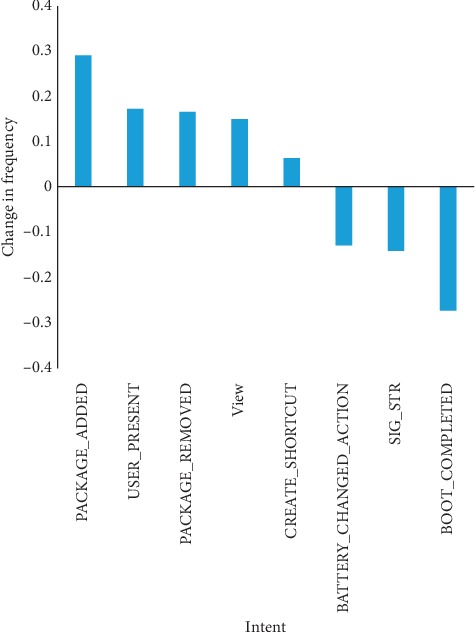
Intent variation frequencies between *Pass* and *New* datasets.

From Figure 11, we find three main differences between *Pass* and *New* datasets. First, permissions for accessing Internet and phone state are the most requested permissions. Second, comparing Android apps from the *New* dataset with those from the *Pass* dataset, we find that the frequency of SMS-related permission reduces. Third, the frequency of related permissions of the device from Android apps of the *New* dataset are more than that of the *Pass* dataset, such as *SYSTEM_ALERT_WINDOW*.  Figure 12 shows the variation of intent.

From the change of intent behavioral characteristics between *New* and *Pass* datasets, we obtain two main conclusions. First, the frequency of intent of monitor phone signal and battery has been reduced. Second, the Android malware from the *New* dataset employs more intent of visibility setting.

## 5. Conclusion

In this paper, we introduce an informative and comprehensive Android malware behavioral characteristics analysis methodology, which aims to detect malicious activities during the data transmission of brain-machine interfacing. To achieve this goal, we first extract two categories of behavioral characteristics from the Android app, namely, static behavioral characteristics and dynamic behavioral characteristics. These kinds of extracted behavioral characteristics are able to cover a majority of Android app behaviors. Then, we explain the role of these kinds of behavioral characteristics play in the Android app analysis and Android malware detection. In the experiment, we design three kinds of evaluations which aim to verify the performance and persistence of extracted behavioral characteristics.

## Figures and Tables

**Algorithm 1:**
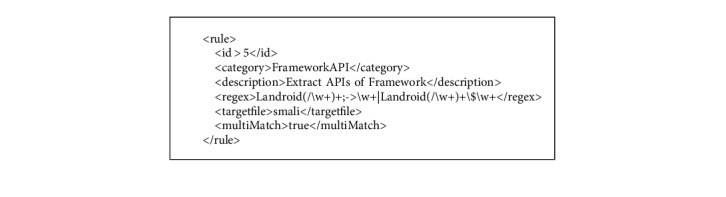
An example of feature extraction rule.

**Table 1:** Some behavioral characteristic instances in *platform-defined characteristic*.

Behavioral characteristic	Instance
Requested permission	ACCESS_GPS, GET_TASK, WAKE_LOCK
Hardware	CAMERA, NFC, GPS
API call	util.log.w, Dialog.show, Uri.prase
Protected API	getDeviceId, sendSMS, getWifiState
Used permission	INTERNET, SEND_ SMS, READ_CONTACT
Code pattern	MessageDigest, loadLibrary, pathClassLoader
Payload information	.MF, .RSA, .jpg

**Table 2:** Some behavioral characteristic instances in *app-specific characteristic*.

Behavioral characteristic	Instance
App component	com.google.search, com.eguan.state, com.google.update
Intent	PHONE_STATE, MAIN, SIG_STR
String	map.Google.com, http://www.umeng.com, media. admob.com
Certificate information	2b7172a335b66873dc793af3fe5c3fc6d8…, 5fb16d12bc8a36b9071907bc6e042840c2…

**Table 3:** Performance of single behavioral characteristic.

Behavioral characteristic	Algorithm	TP	FP	Precision	Recall	F-measure	ROC area
Code	SVM	0.685	0.318	0.686	0.685	0.685	0.684
	KNN	0.692	0.324	0.692	0.692	0.689	0.734
	Random forest	0.694	0.323	0.694	0.694	0.69	0.735
Hardware	SVM	0.548	0.535	0.563	0.548	0.411	0.507
	KNN	0.548	0.534	0.564	0.548	0.413	0.543
	Random forest	0.548	0.534	0.564	0.548	0.413	0.547
Indent	SVM	0.689	0.489	0.762	0.689	0.723	0.69
	KNN	0.836	0.184	0.849	0.836	0.833	0.885
	Random forest	0.84	0.181	0.854	0.84	0.837	0.892
Requested permission	SVM	0.873	0.137	0.876	0.873	0.872	0.868
	KNN	0.936	0.064	0.936	0.939	0.936	0.972
	Random forest	0.937	0.063	0.938	0.937	0.937	0.975
Suspicious API	SVM	0.753	0.0.257	0.752	0.753	0.752	0.749
	KNN	0.782	0.228	0.782	0.782	0.781	0.861
	Random forest	0.779	0.231	0.779	0.779	0.777	0.861
Used permission	SVM	0.837	0.177	0.841	0.837	0.835	0.83
	KNN	0.864	0.138	0.864	0.864	0.864	0.942
	Random forest	0.867	0.133	0.867	0.867	0.867	0.943
API	SVM	0.843	0.125	0.859	0.843	0.851	0.905
	KNN	0.831	0.134	0.895	0.831	0.862	0.919
	Random forest	0.904	0.092	0.916	0.904	0.91	0.927
Payload	SVM	0.555	0.531	0.755	0.555	0.407	0.512
	KNN	0.699	0.313	0.698	0.699	0.697	0.774
	Random forest	0.7	0.311	0.699	0.7	0.698	0.779
Cert	SVM	0.744	0.344	0.796	0.744	0.769	0.8
	KNN	0.764	0.319	0.758	0.764	0.761	0.786
	Random forest	0.807	0.183	0.806	0.807	0.807	0.834
String	SVM	0.724	0.269	0.731	0.724	0.727	0.76
	KNN	0.544	0.544	0.544	0.544	0.544	0.544
	Random forest	0.763	0.32	0.736	0.762	0.763	0.784
Component	SVM	0.839	0.164	0.84	0.839	0.839	0.851
	KNN	0.85	0.192	0.863	0.85	0.856	0.872
	Random forest	0.893	0.117	0.893	0.847	0.87	0.875

**Table 4:** Performance of static behavioral characteristic combination.

Behavioral characteristic set	Algorithm	TP	FP	Precision	Recall	F-Measure	ROC area
Platform-defined	SVM	0.887	0.123	0.892	0.887	0.886	0.88
	KNN	0.94	0.06	0.94	0.94	0.94	0.975
	Random forest	**0.942**	**0.058**	**0.942**	**0.942**	**0.942**	**0.981**
App-specific	SVM	0.898	0.102	0.899	0.898	0.898	0.895
	KNN	0.961	0.039	0.961	0.961	0.961	0.981
	Random forest	**0.961**	**0.039**	**0.961**	**0.961**	**0.961**	**0.99**
All	SVM	0.905	0.095	0.909	0.905	0.904	0.899
	KNN	0.962	0.038	0.962	0.962	0.962	0.981
	Random forest	**0.971**	**0.029**	**0.971**	**0.97**	**0.971**	**0.992**

**Table 5:** Performance of dynamic behavioral characteristic combination.

Behavioral characteristic set	Algorithm	TP	FP	Precision	Recall	F-Measure	ROC area
Quantitative	SVM	0.914	0.086	0.914	0.914	0.914	0.905
	KNN	0.909	0.091	0.908	0.928	0.908	0.889
	Random forest	**0.927**	**0.073**	**0.927**	**0.927**	**0.927**	**0.916**
Timing	SVM	0.875	0.125	0.858	0.875	0.866	0.5
	KNN	0.875	0.125	0.858	0.875	0.866	0.5
	Random forest	**0.881**	**0.119**	**0.881**	**0.881**	**0.881**	**0.59**
Semantic	SVM	0.91	0.09	0.91	0.91	0.91	0.899
	KNN	0.902	0.092	0.902	0.902	0.902	0.881
	Random forest	**0.917**	**0.093**	**0.917**	**0.917**	**0.917**	**0.903**
System call	SVM	0.855	0.145	0.856	0.856	0.856	0.879
	KNN	0.812	0.188	0.815	0.815	0.815	0.846
	Random forest	**0.871**	**0.129**	**0.871**	**0.87**	**0.871**	**0.892**
All	SVM	0.945	0.055	0.945	0.938	0.948	0.966
	KNN	0.947	0.053	0.947	0.942	0.945	0.961
	Random forest	**0.965**	**0.035**	**0.979**	**0.977**	**0.979**	**0.986**

**Table 6:** Performance of the dynamic behavioral characteristic combination.

Dataset	Description
Pass training set	**4,403** Android benign apps from Google play **3,982** Android malware (from Drebin, Android Genome project)
Pass testing set	**4,000** Android apps from third-party markets
New training set 3,948 Android malware	**4,455** Android benign apps from Google play
New testing set	**4,000** Android apps from third party markets

## Data Availability

The data used to support the findings of this study are available from the author upon request (suxin@hnu.edu.cn).

## References

[1] Liang W., Tang M., Long J., Xu J., Li K. C. (2018). A secure FaBric blockchain based data transmission technique for industrial IoT. *IEEE Transactions on Industrial Informatics*.

[2] Li J., Sun L., Yan Q., Li Z., Srisa-an W., Ye H. (2018). Significant permission identification for machine-learning-based android malware detection. *IEEE Transactions on Industrial Informatics*.

[3] Aafer Y., Du W. L., Yin H. (2013). DroidAPIMiner: mining api-level features for robust malware detection in android. *SecureComm*.

[4] Talha K. A., Alper D. I., Aydin C. (2015). Apk auditor: permission-based android malware detection system. *Digital Investigation*.

[5] Arp D., Spreitzenbarth M., Hubner M., Gascon H., Rieck K. Drebin: effective and explainable detection of android malware in your pocket.

[6] Xie N., Wang X., Wang W., Liu J. (2018). Fingerprinting Android malware families. *Frontiers of Computer Science*.

[7] Liang W., Li K. C., Long J., Kui X., Zomaya A. (2019). An industrial network intrusion detection algorithm based on multi-characteristic data clustering optimization model. *IEEE Transactions on Industrial Informatics*.

[8] Peng Y., Lu B.-L. (2017). Discriminative extreme learning machine with supervised sparsity preserving for image classification. *Neurocomputing*.

[9] Enck W., Gilbert P., Chun B. (2014). An information-flow tracking system for realtime privacy monitoring on smartphones. *ACM Transactions on Computer Systems*.

[10] Su X., Liu X., Lin J., He S., Fu Z., Li W. (2017). De-cloaking malicious activities in smartphones using HTTP flow mining. *KSII Transactions on Internet and Information Systems*.

[11] Gorla A., Tavecchia I., Gross F., Zeller A. Checking app behavior against app descriptions.

[12] Yu F., Saswat A., Isil D., Alex A. Apposcopy: semantics-based detection of android malware through static analysis.

[13] Enck W., Octeau D., McDaniel P., Chaudhuri S. A study of android application security.

[14] Yang W., Xiao X. S., Andow B., Li S. H., Xie T., Enck W. Appcontext: Differentiating malicious and benign mobile app behaviors using context.

[15] Zhu H.-J., You Z.-H., Zhu Z.-X., Shi W.-L., Chen X., Cheng L. (2018). DroidDet: effective and robust detection of android malware using static analysis along with rotation forest model. *Neurocomputing*.

[16] Mehtab A., Shahid W. B., Yaqoob T., Abbas H., Afzal H., Saqib M. N. (2019). AdDroid: rule-based machine learning framework for android malware analysis. *Mobile Network and Application*.

[17] Xie L., Li S. (2018). Android malware detection model based on bagging-SVM. *Journal of Computer Applications*.

[18] Yan L. K. Droidscope: seamlessly reconstructing the os and dalvik semantic views for dynamic android malware analysis.

[19] Saracino A., Sgandurra D., Dini G., Martinelli F. (2018). MADAM: effective and efficient behavior-based android malware detection and prevention. *IEEE Transactions on Dependable and Secure Computing*.

[20] Burguera I., Zurutuza U., Nadjm-Tehrani S. Crowdroid: behavior-based malware detection system for android.

[21] Hou S., Saas A., Chen L., Ye Y. Deep4MalDroid: a deep learning framework for android malware detection based on linux kernel system call graphs.

[22] Ali F., Badrul A. N., Rosli S. (2018). Evaluation of network traffic analysis using fuzzy C-means clustering algorithm in mobile malware detection. *Advanced Science Letters*.

[23] APKTOOL, http://ibotpeaches.github.io/Apktool/

[24] An disassembler for androids dex format, http://code.google.com/p/smali/

[25] Felt A. P., Chin E., Hanna S., Song D., Wagner D. Android permissions demystified.

[26] Au K. W. Y., Zhou Y., Huang Z. Pscout: analyzing the android permission specification.

[27] Android.basebridge, http://www.symantec.com/securityresponse/writeup.jsp?docid=2011-060915-4938-99$&$tabid=2

[28] Kang H., Jang J., Mohaisen A. (2015). Detecting and classifying android malware using static analysis along with creator information. *International Journal of Distributed Sensor Networks*.

[29] Zhou Y., Jiang X. Dissecting android malware: characterization and evolution.

[30] Thanh H. L. (2013). Analysis of malware families on android mobiles: detection characteristics recognizable by ordinary phone users and how to fix it. *Journal of Information Security*.

